# Cumulative risk and protection measures data

**DOI:** 10.1016/j.dib.2020.106129

**Published:** 2020-08-05

**Authors:** Bianca C. Bondi, Debra J. Pepler, Mary Motz, Naomi C.Z. Andrews

**Affiliations:** a York University, Department of Psychology, 4700 Keele Street, Toronto, Ontario M3J 1P3, Canada; b Mothercraft, Early Intervention Department, 860 Richmond Street West, Toronto, Ontario M6J 1C9, Canada; c Brock University, Department of Child and Youth Studies, 1812 Sir Isaac Brock Way, St. Catharines, Ontario L2S 3A1, Canada

**Keywords:** Cumulative risk, Cumulative protection, Cross-Domain, Theoretically Grounded, Child maltreatment, Prenatal substance exposure

## Abstract

These data include clinically and theoretically grounded, cross-domain cumulative risk and protection measures. These measures were established for use with three sibling groups at Mothercraft's Breaking the Cycle (BTC), a child maltreatment prevention and early intervention program for substance using mothers and their children. These measures were established using archival data obtained from clients’ charts. The cumulative risk factor measure encompasses: 1) items from a cumulative risk measure utilized in prior BTC research, 2) clinical measures assessing maternal mental health, addiction, and parenting capacity, 3) a measure utilized in studies on adverse childhood experiences, and 4) the Diagnostic Classification of Mental Health and Developmental Disorders of Infancy and Early Childhood (Axis IV: Psychosocial Stressors) [1–3]. The cumulative protection factor measure encompasses: 1) existing early intervention components of services at BTC, 2) clinical measures assessing maternal mental health, addiction, and parenting capacity, and 3) known protective factors outlined in the literature. Both measures were theoretically grounded using the Developmental Model of Transgenerational Transmission of Psychopathology [4], which enabled salient domains of risk and protection to be delineated for children exposed prenatally to substances and accessing child maltreatment prevention and early intervention services. For a description of the process of establishing these measures, the total and cross-domain cumulative risk and protection percentages for the sample, as well as a qualitative interpretation of the balance between domains of risk and protection, see [5]. These measures can contribute to improved future understanding around cumulative risk and cumulative protection in vulnerable populations, salient domains of risk and protection, and the unique interaction that occurs between risk and protective processes in the context of child maltreatment prevention and early intervention.

**Specifications table**

**Table:** 

Subject	Psychology: Clinical Psychology
Specific subject area	Cumulative Risk and Protection
Type of data	Cumulative Risk Measure (table) Cumulative Protection Measure (table)
How data were acquired	Clinical measures, clinical factors, early intervention services, relevant literature
Data format	Raw
Parameters for data collection	Relevant cumulative risk and protection items for children exposed prenatally to substances across theoretically grounded perinatal domains (i.e., mother, other parental figure, family, pre-natal/pregnancy, birth/post-natal, child, parent-child interaction, social network/professional services).
Description of data collection	The cumulative risk measure encompasses prior measures of risk and diagnostic classifications. The cumulative protection measure encompasses components of early intervention, clinical measures, and factors outlined in the literature.
Data source location	Mothercraft's Breaking the Cycle Toronto Canada
Data accessibility	With the article
Related research article	B.C. Bondi, D.J. Pepler, M. Motz, and N.C.Z. Andrews, Establishing Clinically and Theoretically Grounded Cross-Domain Cumulative Risk and Protection Scores in Sibling Groups Exposed Prenatally to Substances, *Child Abuse & Neglect.***108**, 2020 https://doi.org/10.1016/j.chiabu.2020.104631.

## Value of the data

• These data are cumulative risk and protection measures that were clinically grounded in a child maltreatment prevention and early intervention program. Their theoretical foundation enabled a domain-specific conceptualization of risk and protection, which facilitates the consideration of intra- and inter-domain risk and protection. Further, these data enable consideration of cumulative protection in addition to risk.
• Researchers and clinicians interested in understanding cumulative risk and protection across clinically salient perinatal domains in vulnerable children can benefit from these data. The measures take a strength-based approach, highlighting the importance of protection alongside risk. Researchers interested in examining the balance between risk and protection can make use of both measures concurrently.
• These measures can be employed in future validation research in larger samples of vulnerable children. Together, the measures of cumulative risk and protection can contribute to future understanding around how risk and protective processes interact, and highlight salient domains of risk and protection in vulnerable populations.
• Given that these measures were established for use in a highly vulnerable sample of children at Mothercraft's Breaking the Cycle, they are comprehensive measures applicable for use with populations exposed to variable levels of risk.
• These measures can contribute to future research on evidence-based child maltreatment prevention and early interventions that: 1) serve children identified as having high-risk profiles, 2) address the full range of risk factors impacting development, 3) provide individualized interventions specific to vulnerable risk domains, and 4) incorporate the most effective protective factors into practice.

## 1 Data description

Table 1. Cumulative Risk Factor Measure. Dichotomously coded risk factors for substance-exposed children undergoing early intervention organized by perinatal domains (i.e., mother, other parental figure, family, pre-natal/pregnancy, birth/post-natal, child, parent-child interaction, social network/professional services).

**Table 1:** Cumulative risk factor measure.

**DOMAIN**	**SCORING**		
**PARENT - MOTHER**			
Presence of a diagnosed DSM-IV-TR^a^/DSM-5^b^ mental illness	(0) no	unknown	(+1) yes
Family history of mental illness	(0) no	unknown	(+1) yes
Chronic medical illness	(0) no	unknown	(+1) yes
Maternal level of education: has not completed high school	(0) no	unknown	(+1) yes
Conviction history	(0) no	unknown	(+1) yes
Mother has history of child abuse/neglect	(0) no	unknown	(+1) yes
Mother has history of interpersonal violence/trauma	(0) no	unknown	(+1) yes
Maternal anxiety symptoms (clinical level – BAI^c^)	(0) no	unknown	(+1) yes
Mother endorses depressive symptoms (clinical level – CESD-D^d^)	(0) no	unknown	(+1) yes
Teenage parent	(0) no	unknown	(+1) yes
More than 3 births	(0) no	unknown	(+1) yes
Has tried to quit substance use ever	(0) no	unknown	(+1) yes
Reports having had withdrawal symptoms when trying to stop substance use	(0) no	unknown	(+1) yes
Low perceived social support – Family (PSS^e^)	(0) no	unknown	(+1) yes
Low perceived social support – Friends (PSS^e^)	(0) no	unknown	(+1) yes
Is not comfortable with closeness and intimacy (RAAS^f^)	(0) no	unknown	(+1) yes
Does not feel she can depend on others (RAAS^f^)	(0) no	unknown	(+1) yes
Worries about being rejected or unloved (RAAS^f^)	(0) no	unknown	(+1) yes
Low confidence regarding ability to cope with relapse crisis situations (DTCQ^g^)	(0) no	unknown	(+1) yes
History of self-harm behaviours or suicide attempt	(0) no	unknown	(+1) yes
**PARENT - OTHER**			
Secondary parent is absent from child's life	(0) no	unknown	(+1) yes
Presence of a diagnosed DSM-IV-TR^a^/DSM-5^b^ mental illness	(0) no	unknown	(+1) yes
Substance use	(0) no	unknown	(+1) yes
Has tried to quit substance use ever	(0) no	unknown	(+1) yes
Conviction history	(0) no	unknown	(+1) yes
Difficult/dysfunctional or abusive relationship with mother of child	(0) no	unknown	(+1) yes
**FAMILY**			
Maternal pregnancy/Birth of a sibling	(0) no	unknown	(+1) yes
New child adopted	(0) no	unknown	(+1) yes
More than one child in the home	(0) no	unknown	(+1) yes
Medical illness of parent or caregiver	(0) no	unknown	(+1) yes
Death of parent or important person	(0) no	unknown	(+1) yes
Other trauma to significant person in the child's life	(0) no	unknown	(+1) yes
Mother is engaged in a domestically violent relationship/Domestic violence	(0) no	unknown	(+1) yes
Has a primary relationship with substance user	(0) no	unknown	(+1) yes
Parent or caregiver traumatic divorce or separation	(0) no	unknown	(+1) yes
Custody dispute	(0) no	unknown	(+1) yes
New romantic relationship	(0) no	unknown	(+1) yes
New adult in household (e.g., romantic partner)	(0) no	unknown	(+1) yes
Parent or caregiver remarriage	(0) no	unknown	(+1) yes
Substance use by household member (non-parental)	(0) no	unknown	(+1) yes
Parental substance use relapse	(0) no	unknown	(+1) yes
Child protective services involvement	(0) no	unknown	(+1) yes
Removal of non-index child from home	(0) no	unknown	(+1) yes
Child put up for adoption	(0) no	unknown	(+1) yes
Parental unemployment or job instability	(0) no	unknown	(+1) yes
Poverty or near poverty (less than $10,000)	(0) no	unknown	(+1) yes
Head of household has no more than a semiskilled occupation	(0) no	unknown	(+1) yes
Inadequate, unsafe or overcrowded housing or homelessness	(0) no	unknown	(+1) yes
Multiple housing moves (2+)	(0) no	unknown	(+1) yes
Parental arrest	(0) no	unknown	(+1) yes
Parental incarceration (or return from incarceration)	(0) no	unknown	(+1) yes
**PRE-NATAL/PREGNANCY**			
Domestic violence	(0) no	unknown	(+1) yes
Alcohol use	(0) no	unknown	(+1) yes
Cannabis use	(0) no	unknown	(+1) yes
Crack/cocaine use	(0) no	unknown	(+1) yes
Heroin use	(0) no	unknown	(+1) yes
Methadone use	(0) no	unknown	(+1) yes
Other opiates use	(0) no	unknown	(+1) yes
Nicotine use	(0) no	unknown	(+1) yes
Prescription drug use	(0) no	unknown	(+1) yes
Other drug use (eg., amphetamines, hallucinogens, barbiturates/sleeping pills, sedatives/hyponotics/tranquilizers, inhalants	(0) no	unknown	(+1) yes
Poly-substance exposure versus single substance exposure during pregnancy	(0) no	unknown	(+1) yes
Continuous exposure over all three trimesters during pregnancy	(0) no	unknown	(+1) yes
Transiency	(0) no	unknown	(+1) yes
Low maternal weight gain	(0) no	unknown	(+1) yes
High blood pressure/ pre-eclampsia	(0) no	unknown	(+1) yes
Mother overweight pre-pregnancy	(0) no	unknown	(+1) yes
Poor pre-natal nutrition	(0) no	unknown	(+1) yes
Mom >35 years	(0) no	unknown	(+1) yes
Teenage pregnancy	(0) no	unknown	(+1) yes
Minimal prenatal care	(0) no	unknown	(+1) yes
History of miscarriages or terminations	(0) no	unknown	(+1) yes
Diabetes during pregnancy	(0) no	unknown	(+1) yes
Infections/Sexually Transmitted Disease	(0) no	unknown	(+1) yes
Anemia	(0) no	unknown	(+1) yes
Placenta Previa	(0) no	unknown	(+1) yes
Multiple fetuses	(0) no	unknown	(+1) yes
Vaginal bleeding (2nd or 3rd trimester)	(0) no	unknown	(+1) yes
**BIRTH/POST-NATAL**			
Mom >35 years	(0) no	unknown	(+1) yes
Teenage pregnancy	(0) no	unknown	(+1) yes
Caesarean delivery	(0) no	unknown	(+1) yes
Premature delivery	(0) no	unknown	(+1) yes
Birth complications	(0) no	unknown	(+1) yes
Post-partum depression	(0) no	unknown	(+1) yes
Apprehension at birth	(0) no	unknown	(+1) yes
**Post-natal Medical Diagnoses:**			
Fetal Alcohol Spectrum Disorder	(0) no	unknown	(+1) yes
Drug withdrawal	(0) no	unknown	(+1) yes
Genetic disorder	(0) no	unknown	(+1) yes
Seizure/tremors	(0) no	unknown	(+1) yes
Heart complications	(0) no	unknown	(+1) yes
Birth injuries	(0) no	unknown	(+1) yes
Birth defects	(0) no	unknown	(+1) yes
Breathing difficulty	(0) no	unknown	(+1) yes
Low birth weight	(0) no	unknown	(+1) yes
Meconium in placenta	(0) no	unknown	(+1) yes
**Post-natal Interventions:**			
Incubator	(0) no	unknown	(+1) yes
Tube feeding	(0) no	unknown	(+1) yes
Apnea monitor	(0) no	unknown	(+1) yes
Respirator (required ventilation)	(0) no	unknown	(+1) yes
Medication requires	(0) no	unknown	(+1) yes
**CHILD**			
Hospitalization of child	(0) no	unknown	(+1) yes
Child medical illness	(0) no	unknown	(+1) yes
Presence of a diagnosed DSM-IV-R^a^/DSM-5^b^ mental illness	(0) no	unknown	(+1) yes
Presence of Fetal Alcohol Spectrum Disorder diagnosis	(0) no	unknown	(+1) yes
Child in foster care or kin care/Change in primary caregiver	(0) no	unknown	(+1) yes
Child neglect (physical, emotional)	(0) no	unknown	(+1) yes
Child abuse (physical, sexual, emotional)	(0) no	unknown	(+1) yes
Child reunification with parent after separation	(0) no	unknown	(+1) yes
Multiple changes in childcare provider	(0) no	unknown	(+1) yes
Stress surrounding child starting daycare/entered school system	(0) no	unknown	(+1) yes
Challenging temperament style	(0) no	unknown	(+1) yes
**Psychosocial & Health Concerns at Intake:**			
Chronic colds	(0) no	unknown	(+1) yes
Chronic respiratory problems	(0) no	unknown	(+1) yes
Chronic ear infections	(0) no	unknown	(+1) yes
Heart problems	(0) no	unknown	(+1) yes
Gastroenteritis	(0) no	unknown	(+1) yes
Limitation in mobility	(0) no	unknown	(+1) yes
Seizures	(0) no	unknown	(+1) yes
Psychological/emotional problem	(0) no	unknown	(+1) yes
Developmental delays/delays to meet developmental milestones	(0) no	unknown	(+1) yes
Injuries	(0) no	unknown	(+1) yes
Eating problems	(0) no	unknown	(+1) yes
Slow weight gain	(0) no	unknown	(+1) yes
Behind in immunization	(0) no	unknown	(+1) yes
Visual impairment	(0) no	unknown	(+1) yes
Hearing impairment	(0) no	unknown	(+1) yes
Speech impairment	(0) no	unknown	(+1) yes
Cognitive impairment	(0) no	unknown	(+1) yes
Frequent injuries	(0) no	unknown	(+1) yes
Behavioural problems	(0) no	unknown	(+1) yes
Asthma	(0) no	unknown	(+1) yes
**PARENT-CHILD INTERACTION**			
Low parental efficacy (BaP^h^)	(0) no	unknown	(+1) yes
Low parental satisfaction (BaP^h^)	(0) no	unknown	(+1) yes
High defensive responding (PSI^i^)	(0) no	unknown	(+1) yes
High parental distress (PSI^i^)	(0) no	unknown	(+1) yes
High parent-child dysfunctional interactions (PSI^i^)	(0) no	unknown	(+1) yes
Mother's perception of having a difficult child (PSI^i^)	(0) no	unknown	(+1) yes
Clinical level of stress in parenting role (PSI^i^)	(0) no	unknown	(+1) yes
High parental expectations (AAPI^j^)	(0) no	unknown	(+1) yes
Low parental empathy (AAPI^j^)	(0) no	unknown	(+1) yes
Reversed familial/parent-child roles (AAPI^j^)	(0) no	unknown	(+1) yes
High hostile ineffective parenting (NLSCY^k^)	(0) no	unknown	(+1) yes
High inconsistent parenting (NLSCY^k^)	(0) no	unknown	(+1) yes
Low positive parenting (NLSCY^k^)	(0) no	unknown	(+1) yes
Reporting challenging relationship with child	(0) no	unknown	(+1) yes
Child apprehension within first three years of life	(0) no	unknown	(+1) yes
**SOCIAL NETWORK**			
Disadvantaged minority ethnic background	(0) no	unknown	(+1) yes
Immigrant status	(0) no	unknown	(+1) yes
Acculturation or language conflicts	(0) no	unknown	(+1) yes

a Diagnostic and Statistical Manual of Mental Disorders, Fourth Edition, Text Revision; American Psychiatric Association, 2000

b Diagnostic and Statistical Manual of Mental Disorders, Fifth Edition; American Psychiatric Association, 2013

c Beck Anxiety Inventory; Beck, Epstein, Brown, & Steer, 1988

d Center for Epidemiologic Studies Depression Scale; Radloff, 1977

e Perceived Social Support from Friends and Family; Procidano & Heller, 1983

f Revised Adult Attachment Scale; Collins, 1996

g Drug-Taking Confidence Questionnaire; Annis & Martin, 1985

h Being a Parent Scale; Johnston & Mash, 1989

i Parenting Stress Index; Abidin, 1986

j Adult Adolescent Parenting Inventory; Bavolek, 1984

k National Longitudinal Survey of Children and Youth; Statistics Canada, 1994

Table 2. Cumulative Protection Factor Measure. Dichotomously coded protective factors for substance-exposed children undergoing early intervention organized by perinatal domains (i.e., mother, other parental figure, family, pre-natal/pregnancy, birth/post-natal, child, parent-child interaction, social network/professional services).

**Table 2:** Cumulative protective factor measure.

**DOMAIN**	**SCORING**		
**PARENT - MOTHER**			
Attends Basic Life Skills group at BTC^a^	(0) no	unknown	(+1) yes
Attends Emotional Awareness Life Skills group at BTC^a^	(0) no	unknown	(+1) yes
Attends Connections^b^ group at BTC^a^	(0) no	unknown	(+1) yes
Attends Relapse Prevention group at BTC^a^	(0) no	unknown	(+1) yes
Attends Recovery Group at BTC^a^	(0) no	unknown	(+1) yes
Attends Mindfulness group at BTC^a^	(0) no	unknown	(+1) yes
In recovery for substance use	(0) no	unknown	(+1) yes
Attending substance use treatment	(0) no	unknown	(+1) yes
Accessing addiction support	(0) no	unknown	(+1) yes
Accessing urine screens	(0) no	unknown	(+1) yes
Accessing mental health support/therapy/trauma counselling	(0) no	unknown	(+1) yes
High Perceived Social Support – Family (PSS^b^)	(0) no	unknown	(+1) yes
High Perceived Social Support – Friends (PSS^b^)	(0) no	unknown	(+1) yes
Is comfortable with closeness and intimacy (RAAS^c^)	(0) no	unknown	(+1) yes
Feels she can depend on others (RAAS^c^)	(0) no	unknown	(+1) yes
Does not worry about being rejected or unloved (RAAS^c^)	(0) no	unknown	(+1) yes
High confidence regarding ability to cope with relapse crisis situations (old DTCQ^d^)	(0) no	unknown	(+1) yes
Maternal level of education: has completed post-secondary education	(0) no	unknown	(+1) yes
**PARENT - OTHER**			
No substance use history	(0) no	unknown	(+1) yes
If substance use history, in recovery for substance use	(0) no	unknown	(+1) yes
Parent attending substance use treatment	(0) no	unknown	(+1) yes
Accessing addiction support	(0) no	unknown	(+1) yes
Accessing mental health support/therapy/trauma counselling	(0) no	unknown	(+1) yes
Presence of positive secondary parental figure to child	(0) no	unknown	(+1) yes
**FAMILY**			
Partner supportive of maternal substance use treatment services	(0) no	unknown	(+1) yes
Family supportive of maternal substance use treatment services	(0) no	unknown	(+1) yes
Presence of extended familial supports	(0) no	unknown	(+1) yes
High socio-economic status	(0) no	unknown	(+1) yes
Accessing couples therapy services	(0) no	unknown	(+1) yes
Accessing family therapy services	(0) no	unknown	(+1) yes
Family cohesion	(0) no	unknown	(+1) yes
**PRE-NATAL/PREGNANCY**			
Early-intervention through BTC Pregnancy Outreach Program^e^	(0) no	unknown	(+1) yes
Attends BTC Pregnancy Outreach Program^e^ Prenatal Relapse Prevention group	(0) no	unknown	(+1) yes
**BIRTH/POST-NATAL**			
Neonatal follow-up	(0) no	unknown	(+1) yes
**CHILD**			
Easy temperament	(0) no	unknown	(+1) yes
Child was/is in daycare	(0) no	unknown	(+1) yes
Child involved in extra-curricular activities	(0) no	unknown	(+1) yes
Child has positive teacher relationships at school/daycare	(0) no	unknown	(+1) yes
Received occupational therapy	(0) no	unknown	(+1) yes
Received speech/language therapy	(0) no	unknown	(+1) yes
Received psychological assessment	(0) no	unknown	(+1) yes
Child protective services involvement	(0) no	unknown	(+1) yes
**PARENT-CHILD INTERACTION**			
High parental efficacy (BaP^f^)	(0) no	unknown	(+1) yes
High parental satisfaction (BaP^f^)	(0) no	unknown	(+1) yes
Low parental distress (PSI^g^)	(0) no	unknown	(+1) yes
Low parent-child dysfunctional interactions (PSI^g^)	(0) no	unknown	(+1) yes
Mother's perception of having an easy child (PSI^g^)	(0) no	unknown	(+1) yes
Low level of stress in parenting role (PSI^g^)	(0) no	unknown	(+1) yes
Low parental expectations (AAPI^h^)	(0) no	unknown	(+1) yes
High parental empathy (AAPI^h^)	(0) no	unknown	(+1) yes
Intact familial/parent-child roles (AAPI^h^)	(0) no	unknown	(+1) yes
Low hostile ineffective parenting (NLSCY^i^)	(0) no	unknown	(+1) yes
Low inconsistent parenting (NLSCY^i^)	(0) no	unknown	(+1) yes
High positive parenting (NLSCY^i^)	(0) no	unknown	(+1) yes
Attended New Mom Support group at BTC^a^	(0) no	unknown	(+1) yes
Attends Mother Goose group at BTC^a^	(0) no	unknown	(+1) yes
Attends Learning Through Play^j^ group at BTC^a^	(0) no	unknown	(+1) yes
**SOCIAL NETWORK/PROFESSIONAL CARE/SERVICES**			
Non-family adult support network	(0) no	unknown	(+1) yes
Public health services	(0) no	unknown	(+1) yes
High risk nurse services	(0) no	unknown	(+1) yes
Physician	(0) no	unknown	(+1) yes
Financial Allowances (e.g., ODSP^k^, OCCS^l^, Ontario Works)	(0) no	unknown	(+1) yes

a Mothercraft's Breaking the Cycle

b Perceived Social Support from Friends and Family; Procidano & Heller, 1983

c Revised Adult Attachment Scale; Collins, 1996

d Drug-Taking Confidence Questionnaire; Annis & Martin, 1985

e Racine, Motz, Leslie, & Pepler, 2009

f Being a Parent Scale; Johnston & Mash, 1989

g Parenting Stress Index; Abidin, 1986

h Adult Adolescent Parenting Inventory; Bavolek, 1984

i National Longitudinal Survey of Children and Youth; Statistics Canada, 1994

j Pepper & Weitzman, 2004

k Ontario Disability Support Program

l Ontario Child Care Supplement

## 2 Experimental design, materials and methods

The cumulative risk measure was established using items from prior measures, including: 1) items from a cumulative risk measure utilized in prior BTC research, 2) measures used clinically at BTC to assess maternal mental health, addiction, and parenting capacity, 3) a measure utilized in studies on adverse childhood experiences, and 4) the Diagnostic Classification of Mental Health and Developmental Disorders of Infancy and Early Childhood, specifically Axis IV on Psychosocial Stressors [1], [2], [3]. Each risk item is coded dichotomously, with exposure = 1 and no exposure = 0. Risk assignment was intended to be accomplished using statistical criteria (e.g., upper quartile of risk exposure = 1; all others = 0) or *a priori* theoretical and conceptual categorization (e.g., being below the poverty line, single parenthood) and clinical classifications on relevant clinical measures (e.g., clinically significant anxiety), when appropriate.

The cumulative protection measure was established based on 1) existing early intervention components of services at BTC, 2) clinical measures assessing maternal mental health, addiction, and parenting capacity, and 3) known protective factors outlined in the literature. Each protection item is coded dichotomously, with exposure = 1 and no exposure = 0. Again, assignment was intended to be accomplished using statistical criteria (e.g., lower quartile of risk exposure = 1; all others = 0) or *a priori* theoretical and conceptual categorization (e.g., accessing early intervention services), when appropriate.

The sum of the dichotomous elements within each domain can be calculated to yield domain-specific cumulative risk and protection scores. Total cumulative risk and protection scores can be computed by adding the scores across each domain. Total and domain-specific scores can be converted into percentages to ensure that the denominator is dependent on the number of applicable items, with unknown elements removed.

## Ethics statement

Informed consent was obtained from all individual participants included in the study.

## Declaration of competing interest

The authors declare that they have no known competing financial interests or personal relationships which have, or could be perceived to have, influenced the work reported in this article.
